# Identification of Significant Mutations in Spike Protein of SARS-CoV-2 Variants of Concern and the Discovery of Potent Inhibitors

**DOI:** 10.1155/ghe3/5042190

**Published:** 2025-04-28

**Authors:** Mohsen Almakrami, Mohammed Bazuqamah, Mohammed A. Alshehri, Abdulaziz M. S. Alqahtani, Sultan F. Kadasah, Naif Harthi, Rami Ali Alyami, Abdulmajeed Alqurashi, Abdulhadi A. Al Ruwaithi

**Affiliations:** ^1^Department of Pathology and Laboratory Medicine, King Khaled Hospital, Najran 66262, Saudi Arabia; ^2^Department of Medical Laboratory, College of Applied Medical Sciences, Prince Sattam Bin Abdulaziz University, Al-Kharj 11942, Saudi Arabia; ^3^Department of Biology, Faculty of Science, University of Bisha, P.O. Box 551, Bisha 61922, Saudi Arabia; ^4^Emergency Medical Services, Faculty of Applied Medical Sciences, Jazan University, Jazan, Saudi Arabia; ^5^Respiratory Therapy Department, Faculty of Applied Medical Sciences, Jazan University, Jazan, Saudi Arabia; ^6^Department of Biology, College of Science, Taibah University, Madinah 42353, Saudi Arabia; ^7^Department of Clinical Technology, Umm Al-Qura University, Makkah, Saudi Arabia

**Keywords:** mutations, omicron, SARS-CoV-2, spike protein, variant of concern (VoC)

## Abstract

**Background:** SARS-CoV-2 is a positive-sense single-stranded RNA virus that has a propensity for infecting epithelial cells and the respiratory system. The two important proteins, structural and nonstructural proteins, make the architecture of this virus.

**Aim:** This research aimed at studying significant mutations in spike protein of SARS-CoV-2 variants of concern (VoCs) and finding shared mutations among omicron and other four variants (alpha, beta, gamma, and delta). The purpose of this study was to draw structural comparisons between wild type and mutant proteins, followed by identifying potent inhibitors (ligand) that could be used against SARS-CoV-2 spike protein and its latest omicron VoC.

**Methodology:** In this research, we had studied 16 major mutations as well as shared mutations (6) present in spike region of SARS-CoV-2. Subsequently, we determined the structure of the wild-type SARS-CoV-2 protein from the Protein Data Bank (PDB) with the ID 7R4I. Furthermore, the structure of the mutant protein of SARS-CoV-2 omicron variant was modeled in SWISS-MODEL. The ligand dataset for spike protein of SARS-CoV-2 was also collected from literature and different databases. Both wild type and mutant proteins were docked with ligand database in Molecular Operating Environment (MOE). The docking analysis was performed, and two best ligand molecules, AZ_2 and AZ_13, were finalized based on their energy values, interactions, and docking scores to be used against our wild and mutant proteins.

**Results:** AZ_2 demonstrated a docking score of −6.1753 in MOE, with energy values of −4.3889 and −6.1753. It formed key hydrogen bond interactions. AZ_13 showed a docking score of −5.9, with energy values of −9.3 and −5.9, forming hydrogen donor and acceptor interactions with Asp950 (3.06 Å), Ile312 (3.13 Å), and Glu309 (3.27 Å). These interactions suggest strong binding affinity and potential efficacy. Thus, present research work emphasized on identification of significant mutations and finding a potent target-based drug against SARS-CoV-2 and its omicron variant.

**Outcomes:** Based on this computational analysis performed, it is suggested that proposed compound can be used as remedy against SARS-CoV-2 and its omicron variant.

## 1. Introduction

In 1966, Tyrell and Bynoe played a significant role in the identification of coronaviruses, which they identified from individuals who were experiencing the common cold [1]. In December 2019, a cluster of acute atypical respiratory infections was reported in Wuhan, Hubei Province, China, attributed to a novel coronavirus, now classified as severe acute respiratory syndrome coronavirus 2 (SARS-CoV-2). This virus is the etiological agent responsible for coronavirus disease 2019 (COVID-19). This positive-sense single-stranded RNA virus has a propensity for infecting epithelial cells and the respiratory system [2]. COVID-19, like its ancestor SARS-CoV, can cause potentially fatal sickness [3]. Immune responses both innate and adaptive can be triggered by SARS-CoV-2 infection. Consequently, uncontrolled inflammatory innate immune responses, coupled with impaired adaptive immunity, can lead to both localized and systemic tissue damage [4].

The two types of proteins known as structural proteins (SPs) and nonstructural proteins (NSPs) make up SARS-CoV-2 architecture. There are four genes that encode SPs. These genes are named as envelope (E), nucleocapsid (N), membrane (M), and spike (S) [5, 6]. Similarly, the NSPs are primarily functional proteins or enzymes that aid in viral methylation and replication and may trigger host defense mechanisms against infection [7]. SARS-CoV-2's genome contains the S protein, which plays a crucial role in the virus's ability to bind to the angiotensin-converting enzyme 2 (ACE2) receptor and fuse with host cell membranes. It is also essential in triggering the immune response and serves as a key target in the development of potential therapeutic strategies [8]. This protein is a transmembrane trimeric protein with the S1 and S2 subunits. The S1 subunit consists of N-terminal domain (NTD) as well as the receptor-binding domain (RBD) which is crucial for host cell interaction. The S2 subunit facilitates entry of virus and includes two hepta-dimer repeat domains, a fusion peptide domain, an internal fusion peptide, transmembrane domain, and a C-terminal domain [9]. It is important to note that majority of mutations in SARS-CoV-2 occurs in S protein.

The SARS-CoV-2 viral genome's continual evolution gives rise to different variations with a wide variety of mutations. These variations continue to be a major cause of concern as they may decrease vaccine effectiveness and speed up viral transmission. SARS-CoV-2 variants have been divided by Public Health England (PHE) into two categories: variants of interest (VOIs) and variants of concern (VoCs). VOI are variants that have mutations which impair receptor binding capabilities that can enhance transmission rate and reduce the effectiveness of existing therapies and treatments. On the other hand, VoCs refer to a group of variants that already had an impact on disease severity internationally. They have the potential to threaten existing medical practices and vaccinations. These variants also have increased hospitalization rate [10].  Table 1 shows SARS-CoV-2 VoCs and VOI as labeled by the World Health Organization (WHO) [11].

The omicron variant has drawn global attention ever since discovery due to the significant number of mutations that have raised its transmissibility and immune evasion capacity. Basically, the S protein mediates the virus attachment to human cell surface ACE2 receptor, thus facilitating viral entry during infection [12]. Therefore, mutations in the S protein of SARS-CoV-2 variants could significantly influence the structure of the S protein conformation and further the interaction with ACE2 or neutralizing antibodies. The binding affinity between the SARS-CoV-2 S protein and ACE2 receptor is crucial for viral entry into host cells, and alterations in this interaction are linked to increased virulence. Variants of SARS-CoV-2 often carry mutations in the S protein, particularly in the RBD, which enhance ACE2 binding and contribute to greater infectivity and pathogenicity [12].

Several key mutations have been identified that directly impact the S protein's binding properties. For instance, the N501Y mutation, found in the alpha (B.1.1.7), beta (B.1.351), gamma (P.1), and omicron variants, strengthens the interaction between the S protein and hACE2, facilitating more efficient viral entry into host cells. This mutation has been linked to increased transmission rates and infectivity compared to the original wild-type (WT) strain [12]. Similarly, mutations such as L452R and T478K, present in variants like delta and omicron BA.5, further enhance binding affinity while also contributing to immune escape by reducing the neutralization effectiveness of antibodies generated by previous infections or vaccinations [8, 13]. Additionally, structural changes in the S protein's RBD influence other critical interactions, such as the formation of salt bridges and hydrogen bonds with hACE2. For example, the K417 mutation in delta forms a strong salt bridge with hACE2, increasing the virus's ability to bind and infect cells [12]. Moreover, variants such as BA.2 and BA.5, which contain numerous mutations in their RBD regions, have demonstrated even higher binding affinities than earlier strains, contributing to their enhanced transmissibility and pathogenicity.

Inhibitors targeting the SARS-CoV-2 S protein have emerged as a crucial focus in mitigating the virus's ability to infect host cells. These inhibitors are designed to block the interaction between the RBD of the S protein and the human ACE2 receptor, thereby preventing viral entry. A variety of inhibitors have been developed, ranging from small-molecule inhibitors to monoclonal antibodies (mAbs) and peptides, each with varying mechanisms of action [14]. Small-molecule inhibitors are designed to directly bind to the S protein's RBD, effectively disrupting the binding interface with ACE2. For example, compounds such as nafamostat and camostat have demonstrated the ability to inhibit the viral entry process by targeting the S protein's S1/S2 cleavage site, an essential step in viral fusion and entry into host cells [15].

Moreover, mAbs have been one of the most effective therapeutic strategies. Antibodies like casirivimab and imdevimab (Regeneron's cocktail) and bamlanivimab and etesevimab (Eli Lilly's cocktail) bind to distinct regions on the RBD, preventing the S protein from engaging with hACE2 [16]. Peptide-based inhibitors are another promising class of therapeutics, designed to mimic the ACE2 receptor itself and compete for binding with the viral S protein. These peptides effectively block the RBD from interacting with ACE2, thereby neutralizing the virus. For instance, the peptide EK1 has shown broad-spectrum antiviral activity against coronaviruses by targeting the heptad repeat (HR) region of the S protein, preventing the conformational changes necessary for viral fusion [17].

Additionally, antiviral drugs like remdesivir and molnupiravir target viral replication but are often used in conjunction with S protein inhibitors to enhance treatment outcomes [18]. Combinations of mAbs, small molecules, and peptide inhibitors are being investigated to provide a multifaceted approach to combat the virus, especially in light of emerging variants with enhanced resistance to single-drug therapies.

This research particularly focused on studying significant mutations in S protein of SARS-CoV-2 VoCs and finding shared mutations among omicron and other four VoCs (alpha, beta, gamma, and delta) to draw structural comparisons between WT and mutant proteins, followed by identifying potent inhibitors (ligand) against SARS-CoV-2 S protein (both WT and mutant type) and heading toward computational drug design. We performed a detailed computational study to analyze significant mutations found in SARS-CoV-2 VoCs, with an emphasis on the alpha, beta, gamma, delta, and omicron variants. The investigation entailed detecting common mutations in these five VoCs and comparing SARS-CoV-2 protein structures, both WT and mutant, in order to demonstrate structural differences that impact protein function with target proteins, indicating that they might be useful therapies for SARS-CoV-2. This study fills a key gap in our understanding of SARS-CoV-2 suppression and introduces new drugs with promising effectiveness. We recognize that VoC classifications can change prospectively; hence, we indicated that the VoCs investigated were obtained retrospectively. This clarity helps readers comprehend the temporal context of our findings.

## 2. Methodology

**Figure 1:**
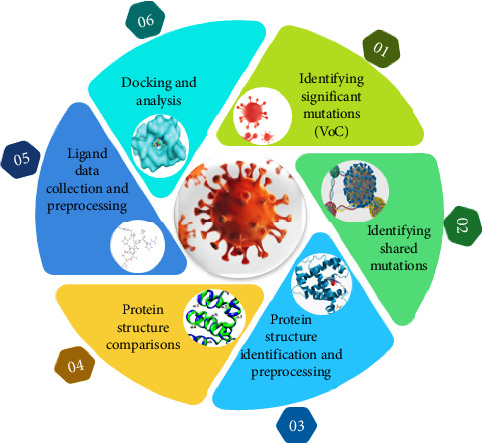
Graphical abstract diagram of methodology followed for work.

In this study, we aimed to identify significant mutations in the S protein of SARS-CoV-2 VoCs and explore potential inhibitors. To achieve this, we employed a combination of computational and experimental approaches. The following section details the methodologies used for data collection, analysis, and validation (Figure 1).

### 2.1. Identification of Significant Mutations in SARS-CoV-2 VoC

**Figure 2:**
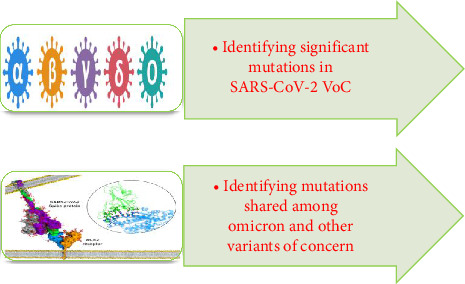
Steps followed during first phase of this work for mutational studies.

The “significance” of mutations was most likely determined by taking into account a variety of parameters, including their frequency in circulating variations, possible influence on viral transmissibility, virulence, or immune response evasion, and relationship with clinical outcomes. These evaluations most likely used a mix of qualitative and quantitative methods to establish the significance of each mutation in the context of SARS-CoV-2 development and its consequences for public health. These significant mutations were studied among different VoCs (alpha, beta, gamma, delta, and omicron). These mutations include deletions and substitutions. Deletion occurs when one or more nucleotides are lost in DNA segment while a substitution mutation switches one base for another. The significant mutations identified among the five VoCs (alpha, beta, gamma, delta, and omicron) include the following: D614G, N501Y, E484K, E484Q, E484A, F486V, L452R T478K, K417N, K417T, T478K, Q493K, Q498R, P681H, P681R, S4779, and ▲69-70 [12, 19]. The steps performed for mutation analysis are given in Figure 2.

### 2.2. Identifying Shared Mutations Among Omicron and VoCs

In this step, a detailed mutational analysis was carried out to find mutations that were common among different VoCs (alpha, beta, gamma, delta, and omicron). The basic purpose was to study the changes that occur in these VoCs with passage of time. These shared mutations were finalized after thorough literature review [9]. The mutations that were shared among omicron and other VoCs include D614G, N501Y, K417N, T478K, P681H, and ▲69-70.

### 2.3. Protein Structure Identification and Preprocessing

#### 2.3.1. WT

After identifying shared mutations among different VoCs, the structure of the target protein, i.e., SARS-CoV-2 S protein, was downloaded from RCSB Protein Data Bank (RCSB PDB) [20]. The PDB ID of the WT protein structure downloaded was 7R4I. The resolution of this structure deposited in PDB was 3.20 Å. The structure with this particular id was named as SARS-CoV-2 S in complex with the 2.15 neutralizing nanobody in PDB [21]. This protein structure was visualized in Molecular Operating Environment (MOE) and was preprocessed to remove extra chains and ligand molecules to prepare it for docking [22].

#### 2.3.2. Mutant Type

To understand the impact of the mutation on structures, 3D structure of a mutant protein is required. The 3D structure for the mutant S protein of SARS-CoV-2 (for shared significant mutations in omicron and other VoCs) was modeled in SWISS-MODEL [23]. SWISS-MODEL is a completely automated protein structure homology-modeling system that may be accessed via the Expasy web server or the software Deep View (Swiss PDB Viewer) [24]. The mutant FASTA sequence of target protein was used to model the structures at default parameters. The two models named mutant 1 and mutant 2 were finalized based on their best statistics which include their sequence coverage and sequence identity. Sequence coverage is the percentage of the query sequence length included in the alignment [25], whereas sequence identity measures the similarity between two sequences [26]. Mutants 1 and 2 were then preprocessed to remove extra chains and ligand molecules because both of them were further to be compared with WT protein. The purpose of this comparison was to observe the structural changes resulting from mutations in these protein structures.

### 2.4. Protein Structure Comparisons

#### 2.4.1. Structural Comparisons of WT Protein With Mutant 1 and Mutant 2

We employed MOE software to analyze and compare the structural differences between mutant and WT proteins. Both the mutant 1 and mutant 2 were compared separately with WT protein in MOE. The purpose of this comparison was to look for structural deviations that occur in mutant structures as a result of significantly shared mutations in S protein of omicron variant. The structures of both WT and mutant proteins were first aligned and then superposed in MOE to check their root mean square deviations (RMSDs). Basically, RMSD is a commonly used metric for comparing two protein structures. The closer two structures are together, the lower the RMSD between them. It actually measures the difference between protein's backbones from their initial structural conformation to their final positions [27]. These superposed structures were then visualized and structural changes were focused. This step was performed to identify the structural changes in both mutant 1 and mutant 2 because small changes in proteins structures can disturb their functioning significantly.

#### 2.4.2. Ligand Data Collection and Preprocessing

Ligand data were collected from various sources including literature and databases. For this particular research, the databases used to collect ligand data were ChEMBL, PubChem, and DrugBank. ChEMBL is a manually curated database of bioactive molecules with drug-like properties [28]. PubChem is the world's largest collection of freely accessible chemical information [29], whereas DrugBank database is also a comprehensive, freely accessible, online database containing information on drugs and drug targets [30]. These ligand data were divided into chemical compounds and phytochemicals.

##### 2.4.2.1. Chemical Compounds

These data of chemical compounds initially downloaded from databases were filtered to remove redundancy in the data. We excluded molecules that had identical ChEMBL IDs and SMILES, inconsistent IC_50_ values, no supporting literature, or were published recently to ensure data reliability, eliminate redundancy, and focus on well-documented, consistent results. After filter application, the remaining compounds were again shortlisted on basis of their IC_50_ values. These IC_50_ values were divided into three classes. These classes include molecules with optimal IC_50_ values, molecules with moderate IC_50_ values, and molecules with high IC_50_ values.

##### 2.4.2.2. Phytochemicals

In addition to chemical compounds, five phytochemicals were also finalized to be used against SARS-CoV-2 S protein from the literature. Next, database of finalized compounds was made in MOE. This database was energy minimized and saved in .mdb format to carry out docking in MOE.

#### 2.4.3. Molecular Docking

The molecular docking was performed in two phases through MOE.

• First, the processed files of both WT protein and ligand database were used as an input for docking.
• Next, the docking was performed with mutant 1 to analyze receptor ligand interactions after mutations in structure.

The triangular matcher method was used at placement phase with London dG scoring function to run docking. In the triangular matcher method, poses were generated by aligning ligand triplets of atoms on triplets of alpha spheres in a more systematic way, whereas the London dG scoring function estimates the free energy of binding of the ligand from a given pose. The number of poses to be generated for each ligand was also specified. The generated poses were further refined in refinement phase. The refinement method used was rigid receptor with scoring function of GBVI/WSA dG. The GBVI/WSA is a forcefield-based scoring function which estimates the free energy of binding of the ligand from a given pose. It has been trained using the MMFF94x and AMBER99 forcefield on the 99 protein-ligand complexes of the SIE training set.

#### 2.4.4. Docking Analysis

The docking results for both wild protein and mutant 1 were visualized in MOE database viewer. The interactions of each pose were observed. From multiple poses generated for individual ligand molecule, one best pose was selected for each molecule by keeping in view their docking score, energy score, and interactions. After analyzing the docking score, energy score, and interactions, two molecules with best statistics were chosen from each class of ligand molecules made previously on basis of their IC_50_ values. These molecules were further reduced from six to two best molecules on basis of their docking score, energy score, and interactions. The purpose was to simplify the analysis. The top two molecules were then finalized from total of six shortlisted molecules on basis of docking scores, interaction, and energy values to be used against our target proteins as best inhibitors for both wild and mutant proteins.

## 3. Results and Discussion

The objective of this research includes finding significant and shared mutations in SARS-CoV-2 VoCs and performing structural comparisons of wild (SARS-CoV-2 S protein) and mutant protein structures (omicron variant) followed by identifying potent inhibitors against SARS-CoV-2 receptor.

### 3.1. Significant Mutations Identified in Structural S Protein of SARS-CoV-2

The SARS-CoV-2 virus was chosen for this research due to the significant mutations occurring in its genome, leading to various changes in the virus itself. In this work, we found sixteen significant mutations in S protein of SARS-CoV-2 from the literature. These sixteen mutations occurred in different VoCs (alpha, beta, gamma, delta, and omicron). From these sixteen mutations identified, five mutations were present in alpha variant, four exists in beta variant, four were present in gamma variant, five were present in delta variant, and ten were present in omicron variant. The significant mutations identified in various SARS-CoV-2 VoCs are represented in Table 2.

In current research, we analyzed all these significant mutations mentioned in Table 2 and their possible effects in S-glycoprotein of five SARS-CoV-2 variants (alpha, beta, gamma, delta, and omicron). In addition, we analyzed the virus binding affinity, transmissibility, virulence, and immune escape patterns that were disturbed as a result of these mutations [33]. The detailed description of these mutations and their impacts are highlighted in Table 3.

### 3.2. Identifying Mutations Shared Among Omicron and Other VoCs

After studying mutations in detail, we found that six mutations out of all sixteen significant mutations present within the S protein of SARS-CoV-2 were shared among the VoCs (alpha, beta, gamma, delta, and omicron). These shared mutations among the VoCs are as follows: D614G, N501Y, ▲69-70, K417N, T478K, and P681H. The D614G mutation is present in alpha, beta, gamma, delta, and omicron variants. The N501Y mutation is shared among alpha, beta, gamma, and omicron variants. The ▲69-70 mutation is present in alpha as well as omicron variant. The K417N mutation is present in beta and omicron variants. The T478K mutation is present in delta and omicron variants. The P681H mutation is shared among alpha and omicron variants.

**Figure 3:**
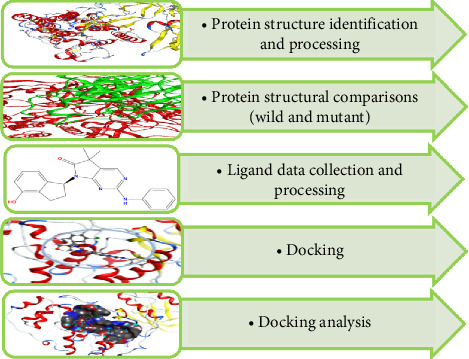
Steps performed during second phase of the study for computational drug design against wild and mutant spike proteins of SARS-CoV-2.

The omicron variant is of particular importance in this study because out of the sixteen significant mutations identified in different VoCs, the omicron variant had the majority of these mutations (i.e., ten). Furthermore, when compared to other VoCs, it contained the most shared mutations. These shared mutations were six in number. These six mutations, which happened separately in other variants, almost all occurred in omicron. For all of these reasons, omicron was chosen for further study. The steps performed in the second phase of the study after detailed mutational analysis are given in Figure 3.

### 3.3. Protein and Ligand Data Collection and Preprocessing

#### 3.3.1. Protein Data Collection and Preprocessing (WT Protein)

We have downloaded structure of target protein, i.e., SPIKE_SARS2 having PDB ID 7R4I (the SARS-CoV-2 S in complex with the 2.15 neutralizing nanobody) from PDB. S protein is classified as viral protein. The sequence length of this protein was 1264. Viruses replicate using the host cell's machinery, but their genetic material encodes the proteins necessary for their life cycle. Despite having fewer genes than cellular organisms, these genes are essential for the replication, assembly, and evasion of host defenses by viruses.

**Figure 4:**
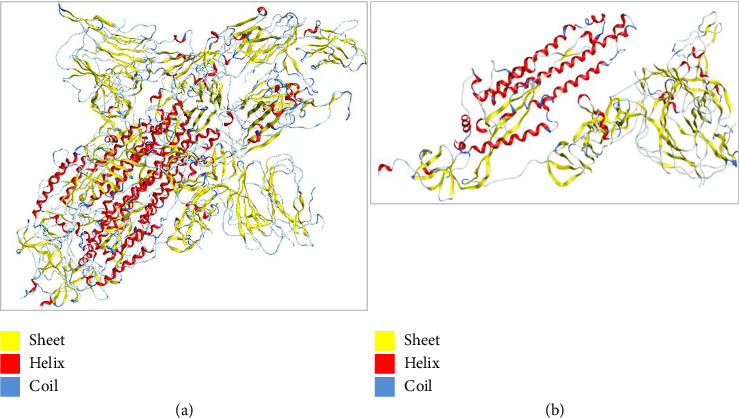
(a) Structure of the wild type protein, i.e., SARS-CoV-2 spike in complex with the 2.15 neutralizing nanobody retrieved from PDB with ID 7R4I. (b) The processed structure of 7R4I visualized in MOE after removing extra chains and ligand molecules. The red color is representing alpha helices, whereas beta sheets are in yellow color and coils and turns are in blue.

To understand the protein geometry, we visualized the structure of protein in MOE. The structure was preprocessed to prepare it for docking. The extra ligand molecules bound to receptor and additional chains B and C that were mirror images of chain A were deleted. The chains D E, F, G, H, I, and J were also deleted because they contained the molecule of no interest, i.e., Camel-derived nanobody 2.15 and 2-acetamido-2-deoxy-beta-D-glucopyranose-(1-4)-2-acetamido-2-deoxy-beta-D-glucopyranose. The structure was protonated to add missing hydrogens if any. The protonated structure was then energy minimized to achieve least energy state (native state of protein, i.e., global minima). Force field used for receptor energy minimization includes Amber 10. The energy minimized structure was saved in .pdb format to perform docking. The structure of the WT protein downloaded from PDB is shown in Figure 4.

### 3.4. Mutant Protein

**Figure 5:**
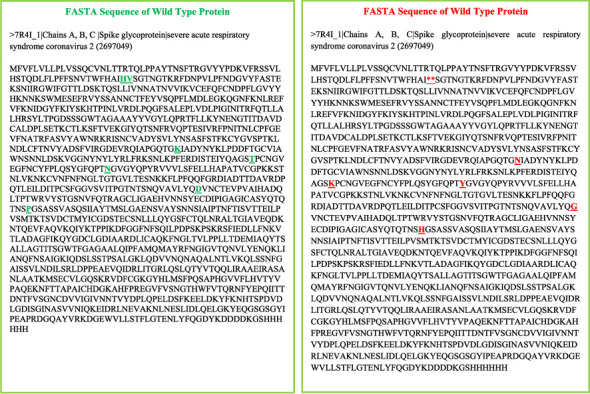
FASTA sequences of wild type and mutant proteins. green is wild type and red is mutant. Amino acid residues in green represents nonmutated state whereas amino acid residues in red represents mutated states.

The mutant structure for omicron S protein of SARS-CoV-2 was modeled from SWISS-MODEL. The mutant sequence with all shared mutations among omicron and other VoCs (alpha, beta, gamma, and delta) was used to model structure. The sequences for WT and mutant protein are given in Figure 5.

In the abovementioned sequences of S protein of SARS-CoV-2 for WT and mutant type, amino acid residues in green represent nonmutated state, whereas amino acid residues in red represent mutated states. These mutated states are either deletion or substitutions. The deleted amino acid residues include H and V, whereas the residues that were substituted include K, T, N, D, and P. These residues were substituted with N, K, Y, G, and H. As a result of these mutations inside the S protein, the host immune system is affected in addition to the virus's ability to replicate and infect new hosts. SARS-CoV-2 S protein mutations have given the virus the capacity to propagate more swiftly and evade the immune response induced by monoclonal neutralizing antibodies or vaccination. The detailed description of these mutations is mentioned in Table 3. Out of 4 models generated from the mutated sequence in SWISS-MODEL, top two models named mutant 1 and mutant 2 were shortlisted for further analysis. Both of these models have maximum similarity of amino acid residues between target-template (sequence identity) as well as highest percentage of query sequence length that is included in the alignment (query coverage). Mutant 1 and mutant 2 are described as follows.

#### 3.4.1. Mutant 1

**Figure 6:**
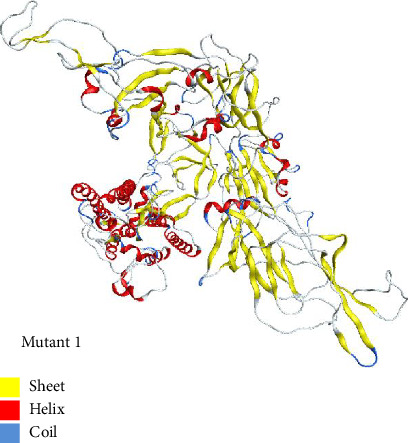
The 3D structure of mutant 1 modeled in SWISS-MODEL for mutant type using the template 7cn8.1 and visualized in MOE.

Mutant 1 was generated in automated mode using template 7cn8.1 (A Glycoprotein Cryo-EM structure of PCoV_GX spike glycoprotein) by SWISS-MODEL. This model had sequence identity of 92.06%. This sequence identity basically highlights target-template alignment. The coverage also lies within range of 1–1028 residues. The 3D structure of mutant 1 is shown in Figure 6.

#### 3.4.2. Mutant 2

**Figure 7:**
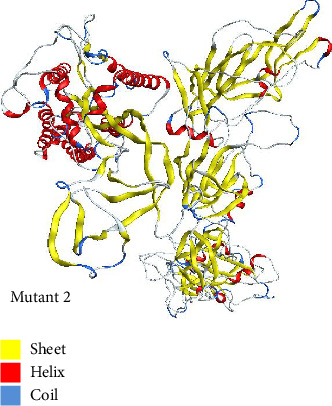
The 3D structure of mutant 2 modeled automatically for mutant type in SWISS-MODEL using the template having PDB ID 6crx.1 and visualized in MOE.

Using template with PDB ID 6crx.1 (SARS Spike Glycoprotein, Stabilized variant, two S1 CTDs in the upwards conformation), mutant 2 was generated automatically in SWISS-MODEL. Sequence identity for this model was 78.93%. The target-template alignment is essentially highlighted by this sequence identity. Additionally, the coverage falls between 1 and 1022 residues. The structure of mutant 2 is shown in Figure 7.

### 3.5. Aligning and Comparing Protein Complexes

The structural comparison of both normal and mutant proteins was performed to visualize the impact of mutations in omicron structure that affects its function. The results highlighted that structural change occurring as a result of these mutations impacts the normal functioning of omicron making it a serious causative agent for COVID-19. Furthermore, the single amino acid substitutions/deletions frequently alter the structure and stability of proteins creating havoc by directly affecting functional binding sites [36]. The result of our analysis also showed significant changes in binding sites of wild and mutant proteins discussed further. These mutational changes in structure of SARS-COV-2 S protein make omicron a significant target in current research. These changes in current study were estimated in terms of RMSD.

### 3.6. Alignment and Comparison of Mutant 1 With WT Protein

**Figure 8:**
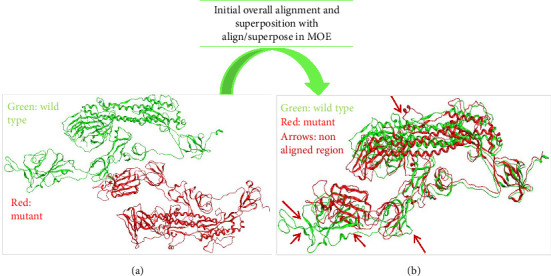
Comparative evaluation of structural changes that occur due to mutations in SARS CoV-2 spike protein observed after aligning and superposing wild and mutant proteins. (a) 3D structures of both wild and mutant 1 proteins as visualized by MOE (green color represents wild type protein and red represents mutant 1 protein). (b) The aligned and superposed geometry of both wild and mutant 1 proteins; the arrows in (b) highlight the nonaligned part between both wild and mutant proteins.

When we aligned mutant 1 with WT S-SARS-CoV-2 protein to measure the similarity between superimposed atomic coordinates of wild and mutant protein chains, the RMSD observed was 11.78Å. Thus, this RMSD value highlights the greater average deviation between the corresponding amino acid residues of both wild and mutant proteins. It means that both of these superposed structures have significant differences in their structures which results from mutations in these structures. As a result of these structural changes in S protein, its normal functioning is disturbed leading toward disturbed immune escape patterns, virus binding affinity, transmissibility, and virulence. The superposed structures of both wild and mutant 1 proteins visualized in MOE are described in Figure 8.

### 3.7. Alignment and Comparison of Mutant 2 With WT Protein

**Figure 9:**
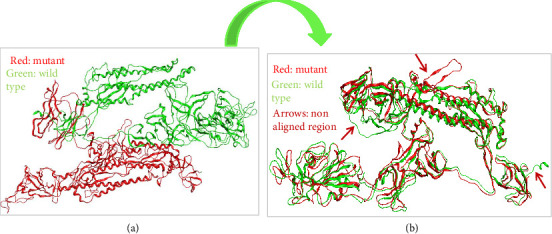
Comparative evaluation of structural changes that occur due to mutations in SARS-CoV-2 spike protein observed after aligning and superposing wild and mutant proteins. (a) 3D structures of both wild and mutant 2 proteins as visualized by MOE (green color represents wild type protein and red represents mutant 2 protein). (b) The aligned and superposed geometry of both wild and mutant 2 proteins; the arrows in (b) highlight the nonaligned part between both wild and mutant proteins.

To check the similarity between mutant 2 and WT protein of S-SARS-CoV-2, we aligned and superposed both of these proteins in MOE. The calculated RMSD for mutant 2 and WT protein was 3 Å. This particular RMSD lies close to acceptable range (≤ 2 Å). This RMSD value is an indication that average deviation between the corresponding amino acid residues of wild and mutant protein is not higher. As a result, the structural changes that occur from mutations in S protein are not much significant. As small change in structure of proteins does affect their normal functioning, these minor changes also contribute toward increased virus infectivity. Surprisingly, various research studies also highlight the importance of structural changes that affect the functioning of protein [36].  Figure 9 represents superposed structures of both mutant 2 and WT proteins as viewed in MOE.

#### 3.7.1. Ligand Data Collection and Preprocessing

The ligand data collected against our wild and mutant proteins was preprocessed to ensure statistical relevance and to remove redundancy by several filter applications. The chemical compounds initially downloaded from ChEMBL, DrugBank, and PubChem databases against our WT and mutant proteins were 50 in number. After preprocessing the dataset (removing the molecules that shared same ChEMBL IDs, similar SMILES, had varying IC_50_ values, had no literature and late publication year), we were left with thirty chemical compounds. These 30 compounds were divided into two classes. Class 1 includes molecules with optimal and moderate IC_50_ values. Class 2 contains molecules with high IC_50_ values. From these 30 compounds, 10 compounds were shortlisted on basis of their IC_50_ values. Class 3 was also made that contains phytochemicals. These phytochemicals includes curcumin, cannabidiol, hesperidin (HD), plitidepsin, and EGCG [37–39]. These phytochemicals also possess varying IC_50_ values. The molecules in these three classes have IC_50_ values in range of tens, hundreds, and thousands. The purpose of choosing molecules belonging to different classes of IC_50_ values was to observe how receptor ligand interaction changes with varying IC_50_ values. The finalized 15 compounds (10 chemical compounds and 5 phytochemicals) with their SMILES and IC_50_ values are represented in Tables 4 and 5.

### 3.8. Docking and Its Analysis

#### 3.8.1. Docking of WT Protein With Ligand Database

Docking is the process of finding the best fit between the receptor and ligand molecules. The docking was performed in MOE to screen the best compound against S protein of SARS-CoV-2 and its omicron variant. The docking results were analyzed in great detail. First, in placement phase, 30 poses were generated for each of the 15 ligand molecules. These 30 poses were further refined in refinement phase. After that, five best poses were specified for individual ligand molecule in refinement phase. Out of five poses generated for each ligand molecule, one best was chosen based on their S score, E score-1, and E score-2. The S score is the final score; E score-1 and E score-2 are the scores from rescoring stages. The poses with the most negative values for S score, E score-1, and E score-2 were selected, as more negative values indicate stronger receptor-ligand attraction and binding. The docking of WT protein with ligands has generated total of 75 poses for 15 ligand molecules. The protein ligand interactions of each finalized pose for every ligand molecule were then analyzed in MOE by viewing 2D interaction diagram. This diagram shows interactions of ligand atoms with binding site residues of receptor molecule.

The number of ligand molecules in docking results was further reduced by dividing the data on basis of their S score, E score-1, E score-2, and receptor ligand interactions. The two ligand molecules with best statistics were chosen from each class of IC_50_, i.e., optimal, moderate, and high. The two molecules having highest energy (most negative energy values), strong interactions, and docking scores were also chosen from phytochemicals. After shortlisting the ligand molecules, we were left with total of six compounds. These six compounds belonged to three classes of IC_50_ values.

Binding energies of the receptor-ligand complex are crucial in docking studies because a higher (less negative) binding energy indicates weaker attraction and binding between the target molecule and the ligand. Furthermore, the most negative values indicate the strong receptor ligand interactions/binding.

#### 3.8.2. Docking Analysis of Class 1 Members

The energy values and docking scores for receptor ligand complexes that were obtained for members of class 1 lie in range of −4.7595 to −6.1753. These scores were obtained as a result of docking in MOE. The docking score and energy values of class 1 members are shown in Table 6.

In Table 6, it is highlighted that among the members of class 1, AZ_2 has the highest docking score and E-score values. The calculated docking score for AZ_2 in MOE was −6.1753. The energy values calculated for AZ_2 were −4.3889 (E-score 1) and −6.1753 (E-score 2). Furthermore, AZ_3 was also another member of class 1 that possesses most negative docking scores and energy values in negative. Thus, the negative values of these two ligand molecules from members of class 1 indicate their strong attraction and binding affinities toward the WT protein.

**Figure 10:**
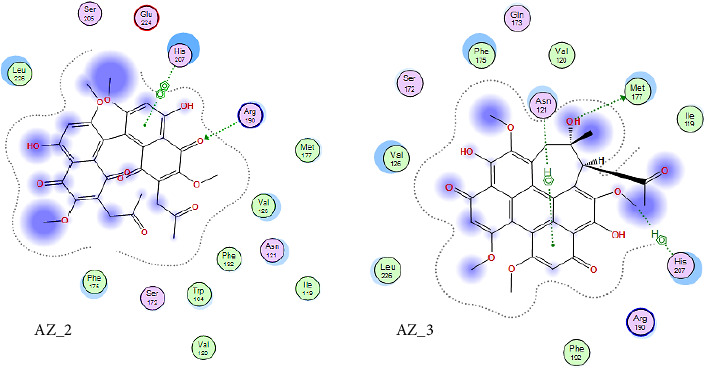
2D interaction diagram of top two ligand molecules, AZ_2 and AZ_3, with receptor SARS-CoV-2 spike protein having PDB ID 74RI visualized in MOE.

The receptor-ligand interaction analysis of the top two ligand molecules from class 1, conducted in MOE, revealed that for molecule AZ_2, the amino acid arginine at position 190 in the receptor interacts with the oxygen atom of the ligand at position 68. This interaction type was H-acceptor which occurs at distance of 2.89. Also, the histidine residue at position 207 interacts with five and six ringed member of ligand forming pi-pi interactions at distance of 3.57. Likewise, the molecule AZ_3 showed three different types of interactions with the WT protein. The first interaction type was H-donor which occurs at distance of 3.83. The amino acid methionine at position 177 in receptor interacts with oxygen atom of ligand molecule at position 55. The second interaction type was H-Pi which occurs at distance of 4.0. The amino acid histidine at position 207 in receptor interacts with oxygen atom of ligand molecule at position 39. The third interaction type was pi-H which occurs at distance of 3.98. The amino acid asparginine at position 121 in receptor interacts with 6-ring member of ligand molecule. Thus, these top two molecules, AZ_2 and AZ_3, were shortlisted from class 1 as the candidates that could be used as potent inhibitors against SARS-CoV-2 S protein. The 2D interaction diagram for AZ_2 and AZ_3 is shown in Figure 10.

#### 3.8.3. Docking Analysis of Class 2 Members

Surprisingly, the analysis of molecules in class 2 revealed that no member of this class had significant interactions with our WT protein. So, no ligand molecule was preferred from this class that could be used as an inhibitor against our target protein.

#### 3.8.4. Docking Analysis of Class 3 Members

The docking studies revealed that for members of class 3, the binding energies and docking scores lie in the range of −4.3480 to 7.0522.  Table 7 displays the class 3 members' docking scores and energy values.

Docking studies had revealed that among five phytochemicals, pilitidepsin and HD showed the best binding with a binding affinity of −7.0522 and −5.9937 kcal/mol with WT protein. When we analyzed the interactions of pilitidepsin with WT protein, it was found that it did not show any favorable interaction with our target protein. Although we did not specifically investigate why pilitidepsin did not attach to the WT SAR-CoV-2 structure in our docking tests, there are a number of possible explanations for this result. Docking findings may be influenced by variations in the viral protein's shape or variances in binding site accessibility. Furthermore, differences in the parameters and scoring methods employed in docking simulations between research studies may have an influence on the results. It is critical to recognize that the success of docking studies might vary depending on a variety of parameters, and our findings do not contradict earlier research. Further research and experimental validation might give further insight into the identified differences, guiding future medication development efforts.

We have considered curcumin for further analysis instead of pilitidepsin from this class along with HD. The energy and docking score for curcumin reported as result of docking was −5.4228.

HD is common flavanone glycoside that was obtained from citrus fruits. Citrus fruits, a frequent ingredient in traditional Asian treatments, are enriched with HD. HD interacts with WT proteins amino acid residue Asp950 at distance of 3.06 showing H-donor interaction. HD also forms H-acceptor interaction with Ile312 of wild protein at 3.13 distances. Furthermore, it also showed H-acceptor interaction with Glu309 at distance of 3.27. Additionally, Cheng et al. [39] also reported in their study that HD and its aglycone, hesperetin, may help fight COVID-19 by preventing the S protein of SARS-CoV-2 from attaching to the human cellular receptor ACE2 and by lowering the expression levels of ACE2 and TMPRSS2. Regardless of the S protein mutation, these effects severely inhibit the SARS-CoV-2 variant's ability to enter cells. HD may therefore be administered as a possible COVID-19 preventive medication. Furthermore, Ansari et al. [40] also concluded in their study the use of HD as a candidate that could be used against S-glycoproteins of SARS-CoV-2 variants.

**Figure 11:**
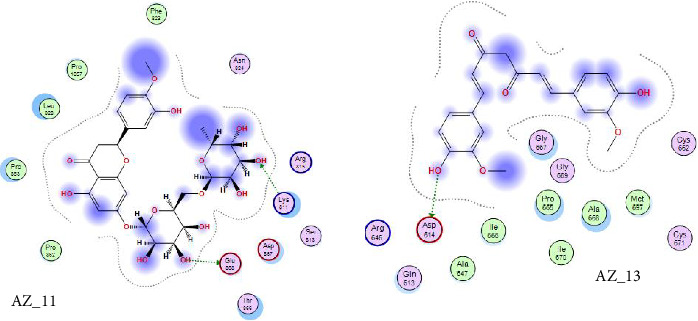
A 2D interaction diagram of the top phytochemicals, AZ_11 and AZ_13, with the wild-type SARS-CoV-2 spike protein (PDB ID: 74RI) was generated and visualized using MOE.

Curcumin is a naturally occurring polyphenolic substance that is isolated from the roots of the *Curcuma longa* that belongs to Zingiberaceae family. It has a variety of medicinal qualities. The turmeric's yellow pigment is used to treat a variety of illnesses caused by inflammation and infection. In our study, it was revealed that for curcumin, the amino acid asparginine at position 614 interacts with the oxygen atom of ligand (curcumin) at position 46. Curcumin showed H-donor interaction with the receptor at distance of 3.11. The authors of [41, 42] also reported in their studies the effectiveness of curcumin as a useful compound to be used against COVID-19 patients. The 2D interaction diagram for AZ_13 and AZ_11 is given in Figure 11.

**Figure 12:**
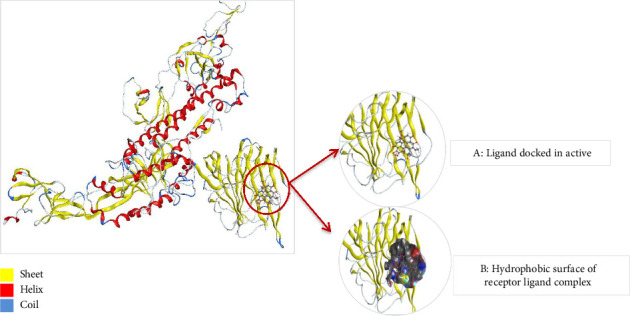
Protein complex after ligand AZ_2 is docked into active site of receptor 7R4I (wild type) and its hydrophobic surface visualized in MOE.

**Figure 13:**
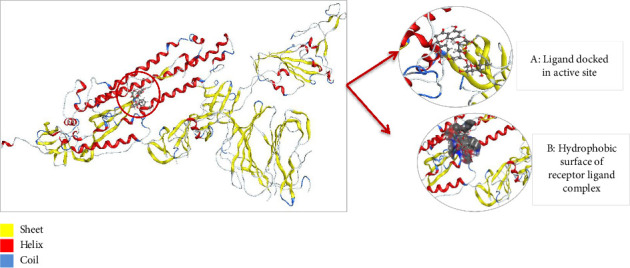
Protein complex after ligand AZ_13 is docked into active site of receptor 7R4I (wild type) and its hydrophobic surface visualized in MOE.

Thus, the docking of members of classes 1, 2, and 3 with WT protein had revealed that the molecules AZ_2, AZ_3, AZ_11, and AZ_13 had shown the best statistics. These statistics include their interactions, binding energies, and docking scores. Furthermore, these four molecules were again shortlisted, retaining only two molecules one from each class. The finalized two molecules that could be used as potent inhibitors against SARS-CoV-2 include AZ_2 and AZ_13. These molecules were shortlisted from 15 molecules because they had shown strong binding energies, docking scores, and attraction toward the target protein. The 3D interaction diagram of both AZ_2 and AZ_13 with receptor 7R4I is shown in Figures 12 and 13.

#### 3.8.5. Docking of Mutant 1 With Ligand Database

Docking of mutant 1 protein of SARS-CoV-2 omicron variant was also performed in MOE. The mutant 1 protein, modeled in SWISS-MODEL, contains all the key mutations found in the Omicron variant, which are also shared by the five major VoCs: Alpha, Beta, Gamma, Delta, and Omicron. The purpose of docking mutant 1 with shortlisted 15 ligand molecules was to check that whether the top two inhibitors (AZ_2 and AZ_13) that showed the most negative energy values, docking scores, and interactions for WT protein also show the most negative values for mutant 1 protein or not. Another aim was to check the interactions of these finalized ligand molecules with mutant 1 to observe their attraction and binding toward the mutant 1 protein. Therefore, all the 15 compounds mentioned in Tables 4 and 5 were again docked with mutant 1 protein in MOE. The mutant 1 protein was chosen for carrying out this docking study instead of mutant 2 because of the greater query coverage and sequence identity values of mutant 1. The docking parameters in MOE were also kept same for mutant protein as they were set for WT protein.

### 3.9. Docking Analysis of Class 1, 2, and 3 Members

The docking results for member of class 1 revealed that for this particular docking of mutant 1 in MOE, AZ_2 had again showed the most negative values of energy and docking scores for mutant 1 protein. The energy values and docking scores for members of class 1 obtained as a result of docking in MOE range from −4.2512 to −6.8870. The docking score and energy values of class 1 members are shown in Table 8.

Table 8 highlights that for mutant 1, AZ_2 had once again shown the most negative value of energy and docking score when compared to other members of same class. The interaction analysis revealed that AZ_2 showed interaction with the amino acid lysine of mutant 1 protein at position 79. The oxygen atom at sixteenth position of ligand atom participates in this interaction. This particular interaction type between receptor ligand complex was H-acceptor. Furthermore, the ligand molecules in class 2 had not shown any significant interactions with mutant protein. Therefore, the final results contain no member from class 2 to be an effective inhibitor against omicron variant of SARS-CoV-2. Similarly, the docking results of phytochemicals with mutant 1 had also shown most negative energy and docking score values for pilitidepsin, but surprisingly, it had not shown any interaction with mutant 1 (reasons already mentioned above in section: Docking Analysis of Class 3 Members), so HD was finalized among members of class 3 to be used against omicron variant of SARS-CoV-2. HD showed pi-H interaction with amino acid leucine of mutant protein at position 100. The 6-ring member of ligand took part in this particular receptor ligand interaction. The docking scores and energy value for class three members are shown in Table 9.

**Figure 14:**
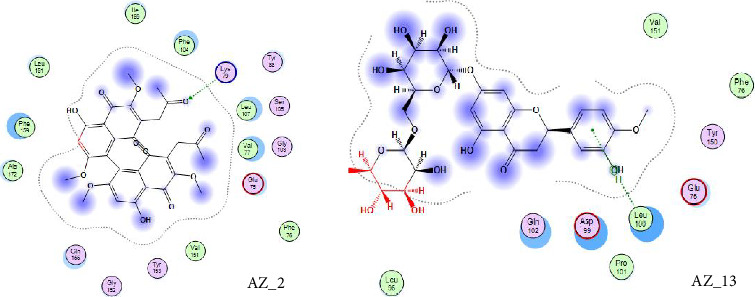
2D interaction diagram of finalized top two ligand molecules, AZ_2 and AZ_13, with receptor omicron variant of SARS-CoV-2 spike protein visualized in MOE.

The 2D interaction diagram for AZ_2 and AZ_13 with mutant 1 visualized in MOE is shown in Figure 14.

**Figure 15:**
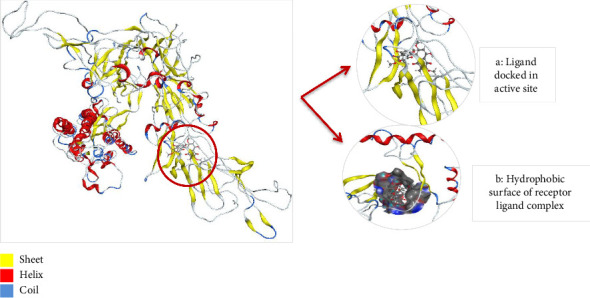
Protein complex after ligand AZ_2 is docked into active site of mutant 1 (a) and its hydrophobic surface (b) visualized in MOE.

**Figure 16:**
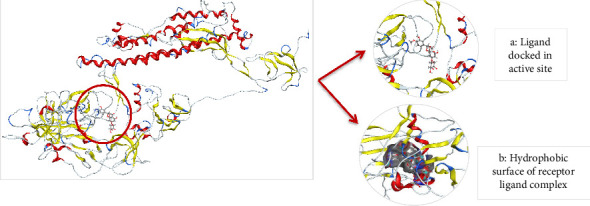
Protein complex after ligand AZ_13 is docked into active site of mutant 1 (a) and its hydrophobic surface (b) visualized in MOE.

The 3D interaction diagram of both AZ_2 and AZ_13 with mutant 1 is shown in Figures 15 and 16.

Thus, after comparing the docking results of both WT and mutant type, we analyzed that the docking results obtained for mutant protein were similar to the docking results of WT protein. Also, the top two finalized inhibitors (AZ_2 and AZ_13) that were proved to be effective inhibitors against SARS-CoV-2 wild protein had shown the most negative energy values and docking scores for mutant protein as discussed above. Therefore, these finalized inhibitors could also be used against SARS-CoV-2 virus when it had attained the stage known as the omicron variant.

## 4. Conclusion

In the current study, detailed computational analysis was carried out where we had identified major mutations present among SARS-CoV-2 VoCs. This study particularly identified shared mutations among five VoCs (alpha, beta, gamma, delta, and omicron). Furthermore, structural comparisons of both wild and mutant protein structures of SARS-CoV-2 were carried out which highlight the structural changes that affect the functioning of these proteins. Next, AZ_2 and AZ_13 were identified as potent inhibitors that could be used for both wild and mutant protein (omicron variant) of SARS-CoV-2. Both of these molecules had shown strong binding and attraction toward wild and mutant proteins of SARS-CoV-2. Based on this computational analysis performed, it is suggested that proposed compound may be a remedy against SARS-CoV-2 both at early onset stage of virus and also when it had attained the most dangerous state particularly the omicron variant stage. Thus, these drug compounds can be used as an effective inhibitor in treating COVID-19 and its complications.

## 5. Recommendations

To advance research on SARS-CoV-2 variants, ongoing surveillance of mutations in the S protein, particularly within the RBD, is essential for maintaining the effectiveness of current treatments and vaccines. Since these mutations can influence viral transmission, immune escape, and treatment resistance, real-time tracking is vital to swiftly address new variants. Inhibitor development should focus on broad-spectrum options that target conserved regions of the S protein, which are less likely to mutate, providing more sustained efficacy across various variants.

## 6. Future Perspective

The results of this in silico study should be validated through experimental methods, including in vitro binding assays, neutralization tests, and in vivo models to confirm the efficacy of the proposed inhibitors. Additionally, molecular dynamics (MD) simulations could be employed in future studies to provide deeper insights into the dynamic behavior and stability of the inhibitor-S protein interactions over time. Combining various therapeutic approaches, such as small molecules, mAbs, peptide inhibitors, and MD simulations, may help overcome resistance and improve overall treatment efficacy by targeting multiple regions of the S protein and refining inhibitor design.

## Figures and Tables

**Table 1:** SARS-CoV-2 VoCs and VOIs reported by WHO.

SARS-CoV-2 (VoCs)	SARS-CoV-2 (VUI)
Alpha	Epsilon
Beta	Zeta
Gamma	Eta
Delta	Theta
Omicron	Kappa

**Table 2:** The significant mutations identified in the spike protein of SARS-CoV-2 variants of concern (alpha, beta, gamma, delta, and omicron).

Mutations	Region in spike protein	Variant of concern (VoC)					References
		Alpha	Beta	Gamma	Delta	Omicron	
D614G	Non-RBD	✓	✓	✓	✓	✓	[12, 31]
N501Y	RBD	✓	✓	✓	—	✓	[12, 13, 32]
E484K	RBD	✓	✓	✓	—	—	[9, 12]
E484Q	RBD	—	—	—	✓	—	[9]
E484A	RBD	—	—	—	—	✓	[31]
L452R T478K	RBD	—	—	—	✓	—	[9, 32]
▲69-70	NTD	✓	—	—	—	✓	[9, 31, 33]
K417N	RBD	—	✓	—	—	✓	[32, 33]
K417T	RBD	—	—	✓	—	—	[12, 31]
T478K	RBD	—	—	—	✓	✓	[12, 32]
Q493K	RBD	—	—	—	—	✓	[9, 31]
Q498R	RBD	—	—	—	—	✓	[9, 32]
P681H	Near furin cleavage	✓	—	—	—	✓	[12, 33, 34]
P681R		—	—	—	✓	—	[12, 34]
S4779	RBD	—	—	—	—	✓	[31, 33]

**Table 3:** The description and possible effect(s) caused by the significant mutations identified in SARS-CoV-2 VoCs.

Mutations	Possible effect(s) of the mutation	References
D614G	One of the earliest discovered and most common mutations is called D614G, in which the aspartic acid (D) at position 614 is mutated to glycine (G). This mutation boosts virulence, ACE2 binding, virus transmission, immune evasion, and spike density. Numerous cell types, particularly colon cells, lung, and liver showed enhanced transduction in response to the D614G mutation. Additionally, it resists proteolytic cleavage better. This makes it 4–9 times highly contagious, although it is not an escape mutation.	[8, 12]
N501Y	In this mutation, asparagine N at position 501 is replaced by tyrosine Y. This mutation results in higher ACE2 binding affinity, immunological evasion, transmissibility, virulence, increased viral proliferation, and tighter spike-ACE2 contact brought on by a conformational shift.	[12, 19]
E484K E484Q E484A	These mutations occur when glutamic acid (E) at position 484 is replaced to either lysine (K), glutamine (Q), or proline (P). The convalescent serum neutralization process has been demonstrated to be significantly impacted by these mutations, which has also been linked to reinfections and vaccine inefficiency. These alterations tighten the spike-ACE2 connection, which is caused by a conformational shift which results in increased antibody escape activity.	[9, 35]
L452R	This mutation occurs when amino acid leucine (L) at position 452 is substituted to arginine (R). Increased infectiousness, increased ACE2 binding affinity, increased virus transmissibility, and immune evasion are the outcomes of L452R mutation. This mutation made the virus less resistant to antibody neutralization and made it more contagious.	[8, 19]
T478K	This mutation occurs when amino acid threonine (T) at position 478 is substituted to lysine (L). The T478K mutation is unique to the delta version. The epitope area, which may interact with neutralizing mAbs, is linked to this mutation. It was also observed that T478K is connected to antibody escape and is close to the mutation E484K. Additionally, the binding and transmissibility of ACE2 are increased by this mutation. The T478K mutation's phenotypic implications are still being investigated; however, it is thought to have an influence on viral infectivity and immune evasion.	[13]
▲69-70	This is a deletion mutation where amino acid residues histidine (H) and valine (V) at position 69 and 70 are deleted. Enhanced resistance to several NTD-directed mAbs, increased virus infectivity, and immune evasion are the effects of this mutation.	[9, 35]
K417N K417T	These mutations occur when lysine (K) at position 417 is replaced to either asparagine (N) or threonine (T). These mutations particularly result in increased virulence, higher ACE2 binding affinity, transmissibility, and immune evasions. It also affects the efficiency of vaccines and reinfections.	[12]
Q493K Q498R	These mutations occur when glutamine (G) at position 493 is replaced to either lysine (K) or arginine (R). These alterations increase the RBD-ACE2 binding affinity by doubling the electrostatic potential. Additionally, immune evasion results from structural changes brought on by amino acid substitutions.	[32]
P681H P681R	These mutations occur when variation at position 681 of the spike protein occurs. Basically, amino acid proline at position 681 is substituted to histidine (H) or arginine (R). The p.681 mutations are thought to be linked to increased furin cleavage at the unfavorable S1/S2 junction region during fusion of cell membrane of virus and host-cell. It leads to increased virus transmissibility.	[12, 35]
S477G	These mutations occur when amino acid change occurs at position 477 in spike protein of SARS-CoV-2. Basically substitution of serine to glycine and asparginine takes place. These mutations result in higher binding affinity for ACE2. The enhanced infectivity, transmissibility, covalent sera, and vaccination escape are also outcomes of these mutations.	[31]

**Table 4:** Chemical compounds with their SMILES and IC_50_ values to be used for docking against wild and mutant proteins of SARS-CoV-2.

Chemical compounds		
Name	SMILES	IC_50_ (nm)
*Class 1 chemical compounds with low and moderateIC*_50_ *values*		
AZ_1	COc1c2c3c4c(c(OC)c(=O)c5c(O)cc(OC)c(c6c(OC)cc(O)c(c1=O)c63)c54)[C@@H](C(C)=O)[C@@](C) (O)C2	38
AZ_2	COC1=C(CC(C)=O)C(=O)c2c(c(O)cc(OC)c2c2c(OC)cc(O)c3c2C(=O)C(CC(C)=O) = C(OC)C3=O)C1=O	170
AZ_3	COc1c(O)c2c(=O)cc(OC)c3c4c(OC)cc(=O)c5c(O)c(OC)c6c(c(c1C[C@](C) (O)[C@H]6C(C)=O)c23)c54	120
AZ_4	CCN(CC)CCCC(C)Nc1ccnc2cc(Cl)ccc12	160
AZ_5	CC(C)C[C@H](NC(=O)CNC(=O)[C@H](CO)NC(=O)[C@H](CO)NC(=O)[C@H](CCC(N)=O)NC(=O)[C@H](Cc1ccc(O)cc1)NC(=O)[C@H](Cc1ccccc1)NC(=O)[C@H](CC(C)C)NC(=O)[C@H](CC(=O)O)NC(=O)[C@H](CCC(=O)O)NC(=O)[C@H](C)NC(=O)[C@H](CCC(=O)O)NC(=O)[C@H](Cc1cnc(nH]1)NC(=O)[C@H](CC(N)=O)NC(=O)[C@H](Cc1ccccc1)NC(=O)[C@H](CCCCN)NC(=O)[C@H](CC(=O)O)NC(=O)[C@H](CC(C)C)NC(=O)[C@H](Cc1ccccc1)NC(=O)[C@@H](NC(=O)[C@H](CCCCN)NC(=O)[C@H](C)NC(=O)[C@H](CCC(N)=O)NC(=O)[C@H](CCC(=O)O)NC(=O)[C@@H](N)CCC(=O)O)[C@@H](C)O)C(=O)NCC(=O)N[C@@H](CCCCN)C(=O)NCC(=O)N[C@@H](CC(=O)O)C(=O)N[C@@H](Cc1ccccc1)C(=O)N[C@@H](CCCNC(=N)N)C(=O)O	100

*Class 2 chemical compounds with highIC*_50_ *values*		
AZ_6	O=C(/C=C/c1ccc(O)c2oc(-c3ccc(O)c(O)c3)cc12)O[C@H](Cc1ccc(O)c(O)c1)C(=O)O	3850
AZ_7	C[C@@H]1O[C@@H](O[C@H]2[C@H](O)[C@@H](O[C@@H]3O[C@@H](C)[C@H](O) [C@@H](O)[C@H]3O)[C@H](O[C@H]3CC[C@@]4(C)[C@@H](CC[C@]5(C)[C@@H]4CC=C4[C@@H]6CC(C) (C)CC[C@]6(C(=O)NCCc6ccccc6)CC[C@]45C)C3(C)C)O[C@@H]2CO)[C@H](O)[C@H](O)[C@H]1O	7370
AZ_8	COc1ccc(NC(=O)[C@]23CCC(C) (C)C[C@H]2C2=CC[C@@H]4[C@@]5(C)CC[C@H](O[C@@H]6O[C@H](CO)[C@@H](O[C@@H]7O[C@@H](C)[C@H](O)[C@@H](O)[C@H]7O)[C@H] (O)[C@H]6O[C@@H]6O[C@@H](C)[C@H](O)[C@@H](O)[C@H]6O)C(C) (C)[C@@H]5CC[C@@]4(C)[C@]2(C)CC3)cc1	9970
AZ_9	COc1ccccc1CNC(=O)[C@]12CCC(C) (C)C[C@H]1C1=CC[C@@H]3[C@@]4(C)CC[C@H](O[C@@H]5O[C@H](CO)[C@@H](O[C@@H]6O[C@@H](C)[C@H](O)[C@@H](O)[C@H]6O)[C@H](O)[C@H]5O[C@@H]5O[C@@H](C)[C@H](O)[C@@H](O)[C@H]5O)C(C) (C)[C@@H]4CC[C@@]3(C)[C@]1(C)CC2 IC50	16,750
AZ_10	C[C@@H]1O[C@@H](O[C@H]2[C@H](O)[C@@H](O[C@@H]3O[C@@H](C)[C@H](O)[C@@H](O)[C@H]3O)[C@H](O[C@H]3CC[C@@]4(C)[C@@H](CC[C@]5(C)[C@@H]4CC=C4[C@@H]6CC(C) (C)CC[C@]6(C(=O)Nc6ccccc6)CC[C@]45C)C3(C)C)O[C@@H]2CO)[C@H] (O)[C@H](O)[C@H]1O	18,120

**Table 5:** Phytochemicals with their SMILES and IC_50_ values to be used for docking against wild and mutant proteins of SARS-CoV-2.

Name	Phytochemical	SMILES	IC_50_ (nm)
*Class 3 phytochemicals with varyingIC*_50_ *values*			
AZ_11	Curcumin	COC1=C(C=CC(=C1)C=CC(=O)CC(=O)C=CC2=CC(=C(C=C2)O)OC)O	11,900
AZ_12	Cannabidiol	CCCCCC1=CC(=C(C(=C1)O)C2C=C(CCC2C(=C)C)C)O	1240
AZ_13	Hesperidin	CC1C(C(C(C(O1)OCC2C(C(C(C(O2)OC3=CC(=C4C(=O)CC(OC4=C3)C5=CC(=C(C=C5)OC)O)O)O)O)O)O)O)O	51,500
AZ_14	Pilitidepsin	CCC(C)C1C(CC(=O)OC(C(=O)C(C(=O)NC(C(=O)N2CCCC2C(=O)N(C(C(=O)OC(C(C(=O)N1)NC(=O)C(CC(C)C)N(C)C(=O)C3CCCN3C(=O)C(=O)C)C)CC4=CC=C(C=C4)OC)C)CC(C)C)C)C(C)C)O	4.3
AZ_15	EGCG	C1C(C(OC2=CC(=CC(=C21)O)O)C3=CC(=C(C(=C3)O)O)O)OC(=O)C4=CC(=C(C(=C4)O)O)O	4240

**Table 6:** The docking score and energy values of members of class 1 after performing the docking of ligand molecules with wild type protein of SARS-CoV-2 in MOE.

Docking score and energy values of class 1 molecules			
Name	S-score	E-score 1	E-score 2
AZ_1	−5.8249	−2.8894	−5.8249
AZ_2	**−6.1753**	**−4.3889**	**−6.1753**
AZ_3	**−6.1001**	**−6.7554**	**−6.1001**
AZ_4	−5.9582	−5.7712	−5.9582
AZ_5	−4.7595	−5.8257	−4.7595

*Note:* The bold values indicate the ligands (AZ_2, AZ_3) as best binding ligands.

**Table 7:** The docking score and energy values of phytochemicals after performing their docking with wild type protein of SARS-CoV-2 in MOE.

Docking score and energy values of class 3 molecules				
Name	Phytochemical	S-score	E-score 1	E-score 2
AZ_11	Curcumin	**−5.4228**	**−6.4546**	**−5.4228**
AZ_12	Cannabidiol	−5.0931	−5.4725	−5.0931
AZ_13	Hesperidin	**−5.9937**	**−9.3800**	**−5.9937**
AZ_14	Pilitidepsin	**−7.0522**	**−5.5058**	**−7.0522**
AZ_15	EGCG	−5.2293	−7.6628	−5.2293

*Note:* The bold values indicate the ligands (AZ_2, AZ_3) as best binding ligands.

**Table 8:** The docking score and energy values of members of class 1 after performing the docking of ligand molecules with mutant 1 protein of SARS-CoV-2 omicron variant in MOE.

Docking score and energy values of class 1 molecules			
Name	S-score	E-score 1	E-score 2
AZ_1	−5.5140	−7.7648	−5.5140
AZ_2	**−6.8870**	**−7.9912**	**−6.8870**
AZ_3	−5.7646	−6.0485	−5.7646
AZ_4	−5.0791	−6.0670	−5.0791
AZ_5	−5.2023	−6.9333	−5.2023

*Note:* The bold values indicate the ligands (AZ_2, AZ_3) as best binding ligands.

**Table 9:** The docking score and energy values of phytochemicals after performing their docking with mutant 1 type protein of SARS-CoV-2 in MOE highlighting hesperidin as top compound with most negative energy values and docking scores.

Docking score and energy values of class 3 molecules				
Name	Phytochemical	S-score	E-score 1	E-score 2
AZ_11	Curcumin	−5.6255	−6.9332	−5.6255
AZ_12	Cannabidiol	−5.2193	−6.7007	−5.2193
AZ_13	Hesperidin	**−5.6286**	**−8.8914**	**−5.6286**
AZ_14	Pilitidepsin	−8.9438	−6.86957	−8.9438
AZ_15	EGCG	−5.4154	−8.5374	−5.4154

*Note:* The bold values indicate the ligands (AZ_2, AZ_3) as best binding ligands.

## Data Availability

All data will be freely available upon request.
